# Micro RNAs from DNA Viruses are Found Widely in Plasma in a Large Observational Human Population

**DOI:** 10.1038/s41598-018-24765-6

**Published:** 2018-04-23

**Authors:** Milka Koupenova, Eric Mick, Heather A. Corkrey, Tianxiao Huan, Lauren Clancy, Ravi Shah, Emelia J. Benjamin, Daniel Levy, Evelyn A. Kurt-Jones, Kahraman Tanriverdi, Jane E. Freedman

**Affiliations:** 1 University of Massachusetts Medical School, Department of Medicine, Division of Cardiovascular Medicine, Worcester, MA 01605 USA; 2 University of Massachusetts Medical School, Department of Quantitative Health Sciences, Worcester, MA 01605 USA; 3 Beth Israel Deaconess Medical Center, Cardiovascular Institute, Boston, MA 02215 USA; 4 Boston University School of Medicine, Department of Medicine, Boston, MA 02118 USA; 5 Boston University School of Public Health, Department of Epidemiology, Boston, MA 02118 USA; 6 National Heart, Lung, and Blood Institute, National Institutes of Health (NHLBI) and Boston University’s Framingham Heart Institute, Framingham, MA 01702 USA; 7 Population Sciences Branch, NHLBI, Bethesda, Maryland 20824 USA; 8 University of Massachusetts Medical School, Department of Medicine, Division of Infectious Disease and Immunology, Worcester, MA 01605 USA

## Abstract

Viral infections associate with disease risk and select families of viruses encode miRNAs that control an efficient viral cycle. The association of viral miRNA expression with disease in a large human population has not been previously explored. We sequenced plasma RNA from 40 participants of the Framingham Heart Study (FHS, Offspring Cohort, Visit 8) and identified 3 viral miRNAs from 3 different human *Herpesviridae*. These miRNAs were mostly related to viral latency and have not been previously detected in human plasma. Viral miRNA expression was then screened in the plasma of 2763 participants of the remaining cohort utilizing high-throughput RT-qPCR. All 3 viral miRNAs associated with combinations of inflammatory or prothrombotic circulating biomarkers (sTNFRII, IL-6, sICAM1, OPG, P-selectin) but did not associate with hypertension, coronary heart disease or cancer. Using a large observational population, we demonstrate that the presence of select viral miRNAs in the human circulation associate with inflammatory biomarkers and possibly immune response, but fail to associate with overt disease. This study greatly extends smaller singular observations of viral miRNAs in the human circulation and suggests that select viral miRNAs, such as those for latency, may not impact disease manifestation.

## Introduction

MicroRNAs (miRNAs) are 19 to 24 nucleotide single-stranded noncoding RNA sequences that are present in all multicellular organisms and regulate a vast number of biological processes^1,2^. MicroRNAs can regulate gene expression by binding to messenger RNA (mRNA) of transcribed genes thereby reducing gene expression. With the exception of the seeded sequence of ~6 nucleotides on the 5′ end, miRNAs do not require perfect alignment with the targeted mRNA sequence to achieve their function^2^. As a result, 1 miRNA has the potential to post-transcriptionally regulate up to 300 different mRNA transcripts that may be related to a broad variety of processes^3,4^. Changes in miRNA signatures have been described in a variety of diseases and can associate with increased risk of cardiovascular disease, diabetes or cancer, among many others.

In addition to multicellular organisms, select viruses encode miRNAs that become expressed in target cells^4,5^. These select viruses include most DNA viruses from the *Herpesviridae*^6^, *Polyomaviridae* and *Adenoviridae*^7^ families. Herpes viruses incorporate their DNA into that of the host and are able to establish a lifelong, dormant state that can periodically cause recurrent infection^6^. Studies have shown that, in target cells, changes in expression of different viral miRNAs regulate the lytic or latent cycles of viral replication. Interestingly, plasma analysis of 4 small cohorts (totaling 214 patients) suggests that seropositivity and plasma viral miRNA expression *do not* completely overlap^8^. This observation indicates that viral miRNA plasma expression does not necessarily predict active infection^8^. The potential viral miRNA signatures of chronic infection and overall effect of these viral miRNAs in humans have yet to be established.

Viruses can increase the inflammatory state of the host beyond the target tissue or cell of replication and various viral infections have long been connected to cancer or cardiovascular disease. Infections with DNA viruses such as human cytomegalovirus (HCMV) and Epstein-Barr virus (EBV) have also been associated with severe myocarditis and adverse cardiovascular complications^9–11^. How viruses suppress immunity, affect global inflammation to establish latency and mediate clinical risk is still unclear. As part of the NIH Common Fund-sponsored Extracellular RNA Communication Consortium, we previously reported that, in a large observational cohort, plasma contains a broad number of human extracellular RNA including miRNAs^12^. Further analysis of the plasma RNA sequencing led to the identification of 3 viral miRNAs from 3 different human viruses that were validated by further screening in the remaining 2763 participants of the Offspring cohort. Interestingly, these 3 viral miRNAs, mostly known to control viral latency, associate primarily with inflammatory biomarkers and failed to associate with overt disease risk. Additionally, viral miRNAs in human plasma modestly associated with advancing age and hypertensive treatment.

## Results

### Identification of viral miRNAs in human plasma

Small RNA sequencing of plasma from 40 FHS participants was performed as previously described^13^. After a series of alignments to human miRNA, tRNA, piRNA, and snoRNA, reads that were not mapped to these sequence types were aligned to the available non-human genomes in the exceRpt small RNA-seq Pipeline (www.genboree.org, as of 2015). This led to the identification of 3 viral miRNAs from 3 different viruses having no homology with any human miRNA or other viral miRNA. Viral miRNA homology was assessed using the BLASTN function in miRBase (www.mirbase.org). All viral miRNAs identified in this group were products of DNA viruses from the *Herpesviridae* family (Supplementary Table S1). The presence of these miRNAs was measured in the plasma of the remaining FHS participants (Offspring Cohort, Visit 8, Table 1) using RT-qPCR^13^. The 3 viral miRNAs identified here originated from EBV, Kaposi’s sarcoma herpes virus (KSHV), and HCMV human viruses and their expression is primarily connected to viral latency (Table 2). The 3 viral miRNAs detected had a frequency of 0.7% (hcmv-miR-US25-2-3p) to 27.7% (kshv-miR-K12-6-5p) in the full cohort of 2763 participants (Table 3, Supplementary Table S2). Of note, the RT-qPCR primers detect only mature miRNAs, thus, this does not represent viral particle presence in the circulation^14^. Additionally, as we screened plasma and not serum, plasma viral miRNAs represent circulating miRNA and are not representative of blood cell content.

**Table 1:** Characteristics of the FHS Offspring Cohort (Visit 8, N = 2763).

Age (years)	67 ± 9
Women, n (%)	1499 (54%)
Body Mass Index (BMI)	28 ± 5
Systolic BP (SBP)	129 ± 17
Diastolic BP (DBP)	74 ± 10
Hypertension (HT)	661 (24%)
Coronary Heart Disease (CHD)	286 (10%)
Cancer (any)	441 (16%)
**Biomarkers**	
P-Selectin, ng/ml	41 ± 14
C-Reactive Protein, mg/L	3 ± 7
Tumor Necrosis Factor Receptor II, pg/ml	2687 ± 1123
Intercellular Adhesion Molecule 1, ng/ml	298 ± 102
Interleukin-6, pg/ml	3 ± 3
Monocyte Chemotactic Protein 1, pg/ml	388 ± 141
Osteoprotegerin, pmol/l	5 ± 2
**Concomitant Treatment**	
Regular Aspirin Use	1256 (45%)
Anti-Hypertensive Medication	1466 (53%)
Lipid Lowering Medication	1336 (48%)

Values are presented as mean ± standard deviation (SD) unless N (%) is indicated.

**Table 2:** Functional characteristics in cells of the viral miRNA identified by sequencing in human plasma.

Viral miRNA	Cell	Function	Plasma presence	Ref.
ebv-miR-BART11-5p	B-cell	Targets EBF-1, critical for B-cell germinal center formation, possibly regulating the expression of EBV latency genes	Not known	^23^
kshv-miR-K12-10a-5p/aka kshv-miR-K12-10a*		Not known	Not known	^51^
hcmv-miR-US25-2-3p	fibroblast	Reduces viral replication, and viral titers by downregulation of host’s eIF4A1 and consequent protein translation	Not known	^21,22^

^*^Functional targets for kshv-miR-K12-10a-5p in cells have not been described, contrary to kshv-miR-K12-10a-3p (previously known as kshv-miR-K12-10a) that mediates cytokine secretion, cell survival, and KSHV gene expression^52,53^.

**Table 3:** Viral miRNA expression in the FHS Offspring Cohort (Visit 8, N = 2763) confirmed by qPCR.

Viral miRNA	N	%	Ct Values (mean ± SD)
ebv-miR-BART11-5p	60	2.2%	21.3 ± 1
kshv-miR-K12-10a-5p	325	11.8%	21.2 ± 1
hcmv-miR-US25-2-3p	19	0.7%	21.1 ± 1
*hsa-miR-486-5p*	*2699*	*97*.*7%*	*15*.*89* ± 2

^*^miRNA levels were determined in the plasma of the participants by RT-qPCR. Human miRNA hsa-mir-486-5p Ct values here are used as a reference to compare host vs. viral miRNA Ct values. Of note, miRNAs in this table are detected by High Throughput Fluidic Dynamic Array using **BioMark System instrument** (Fluidigm) and CT values are not comparable to CT values generated by traditional RT-qPCR methods. Maximum Ct values generated by the BioMark System are CT = 23. Anything above 23 is considered not detectable.

### Identification of plasma viral miRNA-blood mRNA co-expression pairs

In cells, viral miRNAs target specific mRNAs depending on their seeded sequence. Whole blood mRNA transcripts from the same FHS participants have been previously characterized^15^ and we sought to establish possible co-expression pairs between circulating viral miRNAs and blood cell mRNAs. Utilizing 17,318 whole blood mRNA transcripts measured from these FHS participants during the same visit and blood draw, we associated human mRNAs to viral miRNA expression. Using continuous analysis for viral miRNA-mRNA pairs and Bonferroni corrected p < 0.05, we identified 2 viral miRNA-mRNA co-expression pairs. Two of the viral miRNAs, kshv-miR-K12-10a-5p and ebv-miR-BART11-5p, co-expressed with genes related to immune response (MIC1) and cell tissue tension and shape (ARHGAP18), respectively (Table 4).

**Table 4:** Co-expression of plasma viral miRNA with whole blood mRNA transcripts assessed using continuous model analysis and a cut off of Bonferroni p < 0.05 corrected for all 17,318 transcripts in 2395 people (FHS, Offspring Cohort, Visit 8).

Viral miRNA	Human mRNA	Transcript ID	Beta	Correlation p-value	Disease associated with gene target	Function of gene target
kshv-miR-K12-10a-5p	MIC1/C18orf8 (Macrophage Inhibitory Cytokine 1)	3781734	0.07	1.61E-06		Colon cancer associated protein
ebv-miR-BART11-5p	ARHGAP18 (Rho GTPase Activating Protein 18)	2973694	0.23	2.74E-06	Penicilliosis	Rho GTPase activating protein that suppresses F-actin polymerization by inhibiting Rho; regulates cell shape, spreading, and migration

### Viral miRNA presence in human plasma associates with circulating inflammatory and prothrombotic biomarkers

Many viral infections induce inflammation and/or thrombosis, both of which are important factors leading to elevated CVD and cancer risk. Thus, we evaluated if the identified plasma viral miRNAs associate with circulating inflammatory or pro-thrombotic biomarkers as measured in the same blood draw. All 3 viral miRNAs associated with at least 1 inflammatory biomarker (Table 5, Supplementary Table S3). Viral miRNAs originating from KSHV (kshv-miR-K12-10a-5p) and HCMV (hcmv-miR-US25-2-3p) were associated positively with the presence of soluble TNF-receptor II (sTNFRII) protein, while interleukin-6 (IL-6) was associated only with hcmv-miR-US25-2-3p. The presence of ebv-miR-BART11-5p was associated with soluble intracellular adhesion molecule 1 (ICAM-1) and hcmv-miR-US25-2-3 was associated with osteoprotegerin. Finally, hcmv-miR-US25-2-3 was associated with changes in pro-thrombotic P-selectin (Table 5; Supplementary Table S3).

**Table 5:** Significant association of viral miRNAs with inflammatory and pro-thrombotic biomarkers in 2763 people (FHS, Offspring Cohort, Visit 8).

Viral miRNA	sICAM1 fold change (95%CI)	sTNFRII fold change (95%CI)	OPG fold change (95%CI)	IL-6 fold change (95%CI)	P-Selectin fold change (95%CI)
ebv-miR-BART11-5p	0.95 (0.93, 0.98), p = 01.0e-04*				
kshv-miR-K12-10a-5p		1.06 (1.02, 1.11), p = 0.002*			
hcmv-miR-US25-2-3p		1.02 (1.01, 1.04), p = 04.0e-04*	1.02 (1.00, 1.03), p = 0.01*	1.01(1.00, 1.03), p = 0.03	0.98 (0.97, 0.99), p = 0.003*

All associations were significant at p < 0.05. Those surviving corrections for multiple comparisons are marked with *. Full results are available in Table S3.

Fold-change values for quantitative measures are for a 1 SD change in that value. Biomarker values were log-transformed for association analyses; P-selectin- platelet selectin; sTNFRII- soluble tumor necrosis factor receptor II (TNFRSF1B); sICAM1- soluble intercellular adhesion molecule 1; Il-6- interleukin 6; OPG- Osteoprotegerin, aka tumor necrosis factor receptor superfamily 11B (TNFRSF11B).

### Viral miRNAs in human plasma modestly associate with age but do not associate with Coronary Heart Disease, hypertension or cancer

Adverse cardiovascular outcomes such as myocarditis or platelet activation have been described in people infected with DNA viruses and the risk of cardiovascular disease increases with age. Here, we sought to determine if the presence of viral miRNA associates with age or disease outcomes. The presence of miRNA, hcmv-miR-US25-2-3p, associated with age (Table 6). Interestingly, in this non-symptomatic cohort, none of the viral miRNAs associated with sex, coronary heart disease, hypertension, or cancer or concomitant medication (Table 6). One exception was viral miRNA hcmv-miR-US25-2-3p which modestly associated with hypertensive treatment (Table 6).

**Table 6:** Association of viral miRNAs in plasma with age and clinical outcomes in 2763 people (FHS, Offspring Cohort, Visit 8).

	ebv-miR-BART11-5p fold change (95%CI), p-value	kshv-miR-K12-10a-5p fold change (95%CI), p-value	hcmv-miR-US25-2-3p fold change (95%CI), p-value
Age (years)	0.99 (0.97, 1.01), p = 0.4	0.97 (0.93, 1.00), p = 0.1	**1**.**02 (1**.**01**, **1**.**03)**, **p = 0**.004*
Sex (women)	1.02 (0.97, 1.07), p = 0.5	1.02 (0.95, 1.09), p = 0.7	1.01 (0.99, 1.04), p = 0.3
Hypertension (HT)	0.97 (0.91, 1.02), p = 0.2	0.96 (0.89, 1.05), p = 0.4	1.00 (0.97, 1.03), p = 0.9
Coronary Heart Disease (CHD)	0.99 (0.92, 1.07), p = 0.8	0.96 (0.86, 1.08), p = 0.5	1.04 (1.00, 1.08), p = 0.1
Cancer (any)	1.03 (0.97, 1.10), p = 0.3	1.08 (0.98, 1.19), p = 0.1	1.01 (0.98, 1.05), p = 0.4
Regular Aspirin Use	1.00 (0.96–1.05), p = 0.9	1.01 (0.94–1.08), p = 0.9	1.00 (0.98–1.03), p = 0.8
Anti-Hypertensive Medication	0.97 (0.92–1.02), p = 0.2	0.95 (0.88–1.02), p = 0.1	1.03 (1.01–1.06), **p = 0**.**01**
Lipid Lowering Medication	0.98 (0.94–1.03), p = 0.5	0.98 (0.92–1.06), p = 0.6	1.00 (0.98–1.03), p = 0.7

Fold-change values for quantitative measures are for a 1 SD change in that value. Associations are considered significant at p < 0.05. Those surviving corrections for multiple comparisons are marked with *. Biomarker values were log-transformed for association analyses.

## Discussion

This is the first study to describe the presence of viral miRNAs from 3 different viruses in human plasma in a large observational cohort and determine their association with inflammatory biomarkers, risk factors and disease. The specific viral miRNAs found are not related to an active infectious state but are predominantly responsible for latency. Using an unbiased sequencing approach and a cohort of 2763 participants from the FHS, we identified variable levels of these circulating viral miRNAs. The presence of these mostly latency-related viral miRNAs in plasma was associated with inflammatory and prothrombotic biomarkers, and 2 of the miRNAs co-expressed with genes related to immunity, tissue tension and cell shape.

In this study, all viral miRNAs originated from DNA viruses from the *Herpesviridae* family and most of them are known to control the latent (dormant) stage of the viral cycle in cells. DNA viruses such as HCMV, EBV and KSHV have the ability to establish a lifelong persistent infection alternating between latent and lytic cycles of viral repication. During latency, there is an absence of disease, lack of viral production in infected cells, and an absence of viral transmission. HCMV viruses establish latency in hematopoietic progenitor cells and cause mild symptoms in immunocompetent organisms^4,16^. EBV and KSHV establish latency predominantly in B-cells^17,18^. EBV is associated with Hodgkin’s and Burkitt’s lymphoma and nasopharyngeal carcinoma, while KSHV causes lymphoma and Kaposi’s sarcoma^19,20^. All of these human DNA viruses can generate miRNAs in their host target cells that mediate viral replication or dormancy. Additionally, these viruses can coexist with the host without causing overt disease. In fibroblasts, the HCMV miRNA, hcmv-miR-US25-2-3p, is able to reduce viral replication and viral titers^21,22^. In B-cells, EBV miRNA, ebv-miR-BART11-5p, affects B-cell germinal center formation, possibly regulating the expression of EBV latency genes^23^. These previous observations support our findings as the FHS cohort is an observational and non-acute population (free of acute infection).

Regardless of the cell from which the viral miRNAs may originate, all 3 miRNAs identified in plasma were associated with the concomitant presence of at least 1 inflammatory biomarker. Two of the viral miRNAs, originating from KSHV and HCMV, associated with elevated levels of sTNFRII in plasma. Soluble TNFRII modulates biological functions of TNF-alpha by competing with cell surface receptors. TNF-alpha is a primary cytokine and levels of sTNFRII shows high accuracy in measuring inflammation and prognosis of disease. It has been postulated that sTNFRII levels are also a useful quantification of the TH1 immune response^24^. One viral miRNA also associated with changes in the levels of sICAM-1. Soluble ICAM-1 is an intracellular adhesion molecule and is released in plasma with increased inflammation and tissue damage. Circulating levels of sICAM1 have not only been associated with coronary heart and vascular disease but with the severity of infectious diseases such as malaria, sepsis, and dengue hemorrhagic fever^25^. Elevated serum levels of sICAM1 have also been associated with immune suppression in patients with chronic liver disease^26^. Osteoprotegerin is a member of the tumor necrosis factor receptor superfamily and was initially discovered as a contributor to bone turnover homeostasis^27^. Interestingly, patients with multiple myeloma have significantly lower levels of osteoprotegerin^28–30^, and viruses such as KSHV are known to modulate osteoprotegerin levels in a COX2-dependent manner^31^. As shown in our findings, hcmv-miR-US25-2-3p significantly associated with osteoprotegerin. In summary, the overall presence of viral miRNAs in this human cohort is associated with a dysregulated inflammatory and prothrombotic plasma profile but is not associated with overt disease, suggesting that the relationship between inflammation and clinical outcome during the viral dormant stage is complex.

As previously mentioned, the presence of viral miRNA in the circulation has been described in studies that included small numbers of cancer, septic or virus infected patients. In 2 small cohorts of septic patients (33 patients without cancer and 66 with cancer), (EDTA) plasma levels of KSHV miRNAs were elevated, and kshv-miR-K12-12 (not detected in our cohort), in particular, exhibited higher levels in patients of African descent^32,33^. EBV miRNAs have also been detected in plasma of patients with chronic lymphocytic leukemia (CLL) and correlate with shorter survival in 2 independent small cohorts^34^. However, the EBV viral miRNA that we detected in the plasma was not present in these leukemic cohorts^34^. In chronic hepatitis B patients, serum presence of 1 HCMV viral miRNA has been suggested as an indicator for effective interferon treatment^35^. From this study, however, it is unclear if HCMV viral miRNA can freely circulate in plasma or if it is part of the cell miRNome released during the coagulation process that occurs with serum collection. Although EBV and KSHV are associated with oncological disease, in our study, we did not find plasma viral miRNAs associated with cancer. The lack of viral miRNA association with cardiac disease or risk factors suggests that, in immunocompetent individuals, DNA viral infections may manipulate the immune system to establish a latent infection without flagrantly influencing cardiovascular disease.

With regard to the role of miRNAs in plasma, it has been established that miRNAs packaged in microvesicles can be transferred to distant cells where they can affect gene expression and modulate functional effects^36,37^. In the case of EBV, it has been shown that viral miRNAs are delivered to uninfected cells through exosome secretion and exert functional repression of targeted mRNA^38,39^. Establishing the specific targets for the miRNAs found in our population is beyond the scope of our study but certainly merits future investigation. Although our findings do not show an association with cardiovascular disease or cancer, this is an important and novel negative observation suggesting that the presence of these viral miRNAs may not always be harmful.

The presence of viral miRNA in a large well-characterized cardiovascular cohort such as the FHS has not been previously described. Additionally, the presence of the 3 viral miRNAs identified in our study has not been previously reported in plasma. A study utilizing a small patient cohort (n = 250) reported an increase of hcmv-miR-UL112 levels in the EDTA-plasma of hypertensive patients^40^. In our study utilizing 2763 patients (using CPD-plasma) of whom 661 were hypertensive, we did not find this association with hypertension. However, hcmv-US25-2-3p mildly associated with hypertensive treatment. Ethnic differences also exist between our studies^40^ that may contribute to a diverse response to viral susceptibility. Genetic polymorphism of viral immune receptors described in Chinese vs. Caucasian cohorts may reflect differences in responses to viral infection and clinical outcome^41^. In our study, hcmv-US25-2-3p associated positively with sTNFRII, OPG, and IL-6 but negatively with prothrombotic P-selectin (platelet-selectin). Incubation of HCMV-infected cells with platelets increases P-selectin secretion at early stages of infection^42^. In our study hcmv-miR-US25-2-3p associated with reduced P-selectin and increased inflammatory biomarkers, suggesting that dysregulation of the host’s hemostatic/immune response may be necessary for efficient latency.

There are limitations to this study. First, we can only identify viral miRNAs that were already recognized and deposited into the Genboree database prior to our analysis. In addition, there is a potential for primer inefficiency in plasma that may lead to the inability to detect viral miRNA levels below the detection threshold. Another important and related technical limitation is the small size of viral miRNAs and their similarity to host miRNA or to the miRNAs of other DNA viruses. Due to this concern, we confirmed through miRBase that there is no sequence similarity between the viral miRNAs and human miRNAs or other human viruses. Limitations of the co-expression model analysis have been previously described^15^ and further work is necessary to confirm mRNA targets, cells of interest and physiological implication for these viral miRNAs. Finally, the FHS population used in this study (Offspring Cohort, Visit 8) is older and of European descent and ongoing studies in our laboratory are exploring the impact of race, ethnicity and age. Additionally, surrogates of cardiovascular disease such as the carotid intima-media thickness (IMT) test were not available from this cohort visit.

In conclusion, this is the first large observational cohort study to identify expression of 3 viral miRNAs from 3 different viruses that have not been previously identified in the circulation. These miRNAs, which are functionally related mostly to latency in cells, associated with inflammatory and thrombotic biomarkers but did not associate with cardiovascular disease or cancer. The novelty of our findings is that overall DNA viral presence may not associate with prevalent disease despite association with inflammatory markers. Further studies including broader, more inclusive populations are necessary to establish viral miRNA signatures for dormant or active infections and their clinical outcomes.

## Materials and Methods

### Study cohort and design

The Framingham Heart Study (FHS) is a community-based, prospective study of cardiovascular disease and its risk factors. Cohorts undergo an examination at the FHS once every ~4–8 years and have been extensively phenotyped over multiple examinations with a wide variety of noninvasive tests. In the present study we used data and plasma samples from the 8^th^ visit of the offspring (and their spouses) of the Original FHS participants (FHS Offspring Cohort). The participants have an extraordinary wealth of clinical data available allowing us to examine the relation of disease and risk factors to gene expression. As previously described, we determined the broadest number of exRNAs in human plasma by performing RNA sequencing on 40 previously stored samples from FHS participants (Offspring Cohort, Visit 8)^13^. We identified 3 viral miRNAs in plasma samples of 40 participants that were evaluated in the entire FHS cohort (n = 2763) by RT-qPCR (see below). Basic characteristics of the full cohort can be found in Table 1.

### Human subjects

The investigations outlined in this manuscript were conducted according to the principles of the Declaration of Helsinki. Studies outlined by the FHS protocol were approved by and carried out in accordance with Boston University Medical Center and by UMass Medical School Institutional Review Boards. All participants provided informed consent and were identified by number and not by name.

### Biomarker assessment

Biomarker levels were measured in the 2763 participants of the FHS cohort. A detailed description for the detection of circulatory biomarkers has been previously reported^43–45^. Briefly, P-Selectin, C-reactive protein (CRP) and osteoprotegerin were detected in plasma^44,45^. Levels of Interleukin-6 (IL6), soluble Intercellular Adhesion Molecule 1 (sICAM1), Monocyte Chemotactic Protein 1 (MCP1) and soluble Tumor Necrosis Factor Receptor II (sTNFRII) were measured in serum^44,45^. Biomarker levels are provided in Table 1.

### Plasma RNA isolation

RNA isolation from plasma was performed as described^13^. Briefly, RNA samples were isolated from 1 mL plasma using a miRCURY RNA Isolation Kit –Biofluids (Exiqon, Denmark). The RNA isolation was carried out via an automated QIAcube system (Qiagen, Germany). RNA samples were eluted in 14 μl and stored at −80 °C.

### Template Preparation for RNA Sequencing

An Ion Chef System, Ion PI Chip Kit v3 and Hi-Q Chef kits were used for template preparation as described^13^. The entire procedure was automated using the Ion Chef System. At the end of the template preparation, loaded PI Chips (Life Technologies, USA) were sequenced^13^. RNA Sequencing was performed on an Ion Proton System^13^ using the Ion PI Hi-Q Chef Kit (Life Technologies, USA).

### Sequencing Data Analysis Using the Genboree Sequencing Pipeline

Detailed procedures for this analysis using the exceRpt tool available on the Genboree Workbench [http://www.genboree.org/] were previously published^13^. After alignment to endogenous sequences and removal of all contaminants with endogenous sequences, the software aligned the remaining sequences to exogenous small RNAs. Reads not mapped to any exogenous small RNAs were aligned again using sRNAbench to the complete set of viral miRNA sequences available in miRBase.

### RT-qPCR for viral miRNAs in plasma

A detailed description of this procedure was previously provided^13^. Briefly, reverse transcription was performed using the miScript SYBR Green PCR Kit (Qiagen, Germany). Viral miRNA primers were purchased from Qiagen (MD, USA). Pre-amplification was done using miScript Microfluidics PreAMP Kit (Qiagen, MD, USA). RT-qPCR was resolved by Dynamic Arrays (Fluidigm, CA, USA) using primers designed by Qiagen (Supplementary Table S2).

### mRNA expression profiling

Whole blood mRNA expression was measured in 2446 participants in the FHS (Offspring Cohort, Visit 8), using Affymetrix exon array ST 1.0 platform, as previously described^46^. This platform included 17,318 mRNA transcripts. A robust multichip analysis (RMA) algorithm was applied using Affymetrix Power Tools (APT) for generation of signal values (i.e., log-2 transformed expression intensity) to yield an initially normalized dataset.

### Statistics

All statistical analyses were performed using STATA 13.0. Descriptive statistics are displayed as mean ± standard deviation (SD) for continuous variables and count (percentage) for categorical variables. For all plasma viral miRNA detection, any miRNA with undetermined Ct values in 23 cycles was considered not present thereby accounting for the detection limit of the BioMark instrument technology (note, this technology is not a traditional qPCR system and it has different detection limits). Ordinary least squares linear regression models were used to test for association with the Ct value of each viral miRNA that was present and each phenotype (i.e. biomarkers, clinical factors, and disease status). The distributions of biomarker assay levels in the restricted sample were not normally distributed and were consequently natural log (ln) transformed for statistical analysis. To account for the number of statistical comparisons conducted, we employed a false discovery rate (FDR = 5%) correction for the number of phenotypes tested (7 biomarkers and 8 clinical factors, Table 1) within each of the viral miRNAs.

### Viral miRNA-mRNA co-expression analysis

Co-expression analysis was performed only in the FHS participants for whom both viral miRNA and mRNA data were available (N = 2395). For each viral miRNA-mRNA pair (4 viral miRNA x 17,318 mRNA), we performed *continuous analysis*: analysis included only samples in which viral miRNAs were expressed. A linear mixed model implemented in “*lmekin”* function of R^47,48^ was used to model mRNA as a response variable and viral miRNA as an independent variable, adjusting for age, sex, technical covariates for mRNA expression profiling measurements described previously^49^, imputed cell counts^49^, and family structure. Benjamini-Hochberg methods were used to calculate FDR or Bonferroni-corrected *P* < 0.05.

### *In silico* prediction of viral miRNA targets

Viral miRNA targets were predicted using the VIRmiRNA database tool (http://crdd.osdd.net/servers/virmirna) by exactly matching the 7mer seeded region of a viral miRNA with the untranslated region and coding region of mRNAs^50^.

### Data availability

The RT-qPCR data described in this manuscript has been deposited in dbGaP, accession number phs000007.v27.p10; the RNA-seq data can be accessed under Jane Freedman at http://genboree.org/exRNA-atlas/exRNA-Grids.rhtml?grid=analysisTable.

## Electronic supplementary material

Supplementary Tables
