# Supplementary material for: Micro RNAs from DNA Viruses are Found Widely in Plasma in a Large Observational Human Population
Source: Sci Rep. 2018 Apr 23;8:6397. doi: 10.1038/s41598-018-24765-6 (PMC5913337; doi:10.1038/s41598-018-24765-6)
Supplement: Supplementary file 1 — Supplementary Tables [file 41598_2018_24765_MOESM1_ESM.docx]

**Micro RNAs from DNA Viruses are Found Widely in Plasma in a Large Observational Human Population**

Milka Koupenova^1*^, Eric Mick^2^, Heather A. Corkrey^1^, Tianxiao Huan^6,7^, Lauren Clancy^1^, Ravi Shah^3^, Emelia J. Benjamin^4,5,6^, Daniel Levy^6,7^, Evelyn A. Kurt-Jones^8^, Kahraman Tanriverdi^1^, and Jane E. Freedman^1^

**Supplementary Tables**

**Supplementary Table S1:** Human Herpesvirus family and viruses (in black) that gave rise to the miRNAs detected in the plasma of the FHS participants (Offspring Cohort, Visit 8); classification and miRNAs.

| **Abbreviation** | **Other names** | **Virus type/Family** | **Host** | **Mature miRNA (#)** | **References** |
| --- | --- | --- | --- | --- | --- |
| HSV1 | Herpes Simplex Virus 1; HHV-1 | DNA; Alpha Herpesvirus | human | 27 | [PMID:20181707; PMID:22661375; PMID:23512275](http://www.ncbi.nlm.nih.gov/pubmed/20181707) |
| HSV2 | Herpes Simplex Virus 1; HHV-2 | DNA; Alpha Herpesvirus | human | 24 | [PMID:20181707; PMID:19889786](http://www.ncbi.nlm.nih.gov/pubmed/20181707) |
| VZV* | Varicella-zoster virus, HHV3 | DNA; Alpha Herpesvirus | human | none | PMID:19889786 |
| EBV | Epstein-Barr virus; HHV4 | DNA; Gamma  Herpesvirus | human | 44 | [PMID:17604727](http://www.ncbi.nlm.nih.gov/pubmed/17604727) |
| hCMV | Human cytomegalovirus;  HHV5 | DNA; Beta  Herpesvirus | human | 26 | [PMID:22013051; PMID:22715351](http://www.ncbi.nlm.nih.gov/pubmed/22013051) |
| HHV-6A & 6B | Roseolovirus; HHV6 | DNA; Beta  Herpesvirus | human | 8 (6B) | [PMID:22114334](http://www.ncbi.nlm.nih.gov/pubmed/22114334) |
| HHV-7 | Roseolovirus;  HHV7 | DNA; Beta  Herpesvirus | human | none known | [PMID:22114334](http://www.ncbi.nlm.nih.gov/pubmed/22114334) |
| KSHV | Kaposi-sarcoma virus; HHV8 | DNA; Gamma  Herpesvirus | human | 28 | [PMID:22114334](http://www.ncbi.nlm.nih.gov/pubmed/22114334) |

***Human HHV3** has no LAT ortholog, it does not encode miRNA and it was not detected in the FHS. Only viruses in black font were detected in the plasma of the FHS participants.

**Supplementary Table S2**: Primers used to screen the FHS cohort by RT-qPCR

| **Viral miRNA** | **Sequence** | **Virus** | **Company** | **TaqMan/**  **SYBR Green** |
| --- | --- | --- | --- | --- |
| ebv-miR-BART11-5p | CAGTTTGGTGCGCTAGTTG | Epstein-Barr Virus (EBV) | Qiagen | SYBR Green |
| kshv-miR-K12-10a-5p | GCTTGGGGCGATACCAC | Kaposi's sarcoma herpesvirus (KSHV) | Qiagen | SYBR Green |
| hcmv-miR-US25-2-3p | CTTGGAGAGCTCCCGCG | Human Cytomegalovirus (HCMV) | Qiagen | SYBR Green |

**Supplementary Table S3**: Values for all association analyses for all biomarkers with viral miRNAs

| **Biomarkers** | **ebv-miR-BART11-5p**  fold change (95%CI),  p-value | **kshv-miR-K12-10a-5p**  fold change (95%CI),  p-value | **hcmv-miR-US25-2-3p**  fold change (95%CI),  p-value |
| --- | --- | --- | --- |
| P-Selectin | 1.01 (0.99, 1.04), p=0.3 | 1.02 (0.99, 1.06), p=0.2 | **0.98 (0.97, 0.99), p=0.003*** |
| CRP | 0.99 (0.97, 1.02), p=0.7 | 1.00 (0.97, 1.04), p=0.9 | 1.01(1.00, 1.02), p=0.1 |
| TNFRII | 1.01(0.98, 1.04), p=0.5 | **1.06 (1.02, 1.11), p=0.002*** | **1.02 (1.01, 1.04), p=4.0e-04*** |
| ICAM-1 | **0.95 (0.93, 0.98), p=01.0e-04*** | 0.98 (0.95, 1.02), p=0.3 | 1.00 (0.98, 1.01), p=0.5 |
| IL-6 | 0.98 (0.96, 1.01), p=0.2 | 1.00 (0.97, 1.04), p=0.8 | **1.01(1.00, 1.03), p=0.03** |
| MCP1 | 0.99 (0.96, 1.01), p=0.4 | 0.98 (0.95, 1.02), p=0.4 | 1.01(1.00, 1.02), p=0.2 |
| OPG | 1.00 (0.97, 1.02), p=0.8 | 0.98 (0.94, 1.01), p=0.2 | **1.02 (1.00, 1.03), p=0.01*** |

Fold-change values for quantitative measures are for a 1 SD change in that value. Associations are considered significant at p<0.05. Those surviving corrections for multiple comparisons are marked with ****. Biomarker values were log-transformed for association analyses. Abbreviations: P-selectin- platelet selectin; CRP-C-reactive protein; sTNFRII-soluble Tumor Necrosis Factor Receptor II (TNFRSF1B); sICAM1- soluble Intercellular Adhesion Molecule 1; IL6-interleukin 6; MCP1- Monocyte Chemotactic Protein 1: OPG- Osteoprotegerin, aka Tumor Necrosis Factor Receptor Superfamily 11B (TNFRSF11B).
